# Bacterial community structure of *Physalis peruviana* L. fruit exocarp and the presence of pathogens with possible implications on food safety

**DOI:** 10.3389/fpls.2024.1410314

**Published:** 2024-07-18

**Authors:** Gabriela N. Tenea, Diana Molina

**Affiliations:** Biofood and Nutraceutics Research and Development Group, Faculty of Engineering in Agricultural and Environmental Sciences, Universidad Técnica del Norte, Ibarra, Ecuador

**Keywords:** cape gooseberry, bacterial diversity, pathogens, *Candidatus Liberibacter*, *Fusobacterium necrophorum*

## Abstract

**Introduction:**

Cape gooseberry (*Physalis peruviana* L.) is a wellconsumed crop in Ecuador, whose fruits are abundant in bioactive molecules. Its rapid post-harvest deterioration and safety limit its market potential.

**Methodology:**

To gather baseline data on the prevalence of bacterial taxa among groups, we employed 16S ribosomal RNA (16S rRNA) amplicon gene sequencing to detect changes in the bacterial community structure in cape gooseberry fruits harvested from an organic farm production system (# 270 samples x two ripeness stages), and fruits obtained from an open-air market (#270).

**Results:**

This is the first report of bacterial taxa inhabiting cape gooseberry fruits. Shannon’s diversity index revealed that the fruits purchased from the market and the unripe stage had the highest level of bacterial diversity (average Shannon indices of 3.3 and 3.1) followed by those collected from the field at the mature ripe stage (2.07). Alpha diversity analysis indicated that there were no significant differences in the number of taxa or evenness within the sample, whereas there was a significant difference in beta diversity between the groups. *Rhizobiaceae* was the most abundant family in fruits originating from the field regardless of the ripe stage, while *Acetobacteraceae*, *Pseudomonadaceae*, *Fusobacteriaceae*, *Bacteroidaceae*, and *Erwiniaceae* were the most abundant families in the market group. At the genus level, *Liberibacter* was the most abundant phytopathogen in fruits originating from the field, while *Gluconobacter* was the most abundant in samples collected from the market. The phytopathogen *Candidatus_Liberibacter* was the most abundant in samples collected from the field, while the fruits purchased from the market stands contained opportunistic enteric pathogens such as *Escherichia vulneris*, *Klebsiella pneumoniae*, and *K. variicola*, their relative abundance varied with the sample. In addition, potential pathogens of animal origin such as *Fusobacterium necrophorum*, *Porphyromonas levii*, *Helcococcus ovis*, and *Trueperella pyogenes* were found in almost all samples at varying relative abundance.

**Conclusion:**

Our study provides basic information on the microbiome of cape gooseberries from agriculture fields to the table along with the detection of several pathogenic microorganisms with possible impact on food safety and public health therefore, strategies for reducing bacterial contamination in both farm and retail markets are compulsory.

## Introduction

*Physalis peruviana* L. known as the cape gooseberry (uvilla or uchuva) is a perennial plant of the *Solanaceae* family that originates from the Andean region. The plant grows at high elevations (above 2000 m above sea level) and Ecuador is a perfect place to cultivate. The fruit and vegetable sector in Ecuador has shown an increase in its participation, contributing 16% to the country’s agricultural GDP, this without considering the production of potatoes and bananas (Moreno-Miranda et al., 2019). Unlike Colombia where this crop is grown traditionally, being the second most exported fruit, Ecuador is still in the incipient phase. Several plantations in the provinces of Pichincha, Carchi, and Imbabura cover 74% of the total area of this fruit in the country. With production ranging from 104 to 350 tons/ha, the province of Imbabura (northern Ecuador) accounts for 40% of the total organic gooseberry production in the country (AGROCALIDAD, 2023). Ecuador reported that cape gooseberries have high potential in the international market receiving $12,294 per ton (Central Bank of Ecuador, 2023). Fruits are rich in vitamins (vitamin C, E, K1 and vitamin B complex) and a variety of bioactive molecules, including phosphorus, iron, potassium, and zinc (Puente et al., 2011; Kasali et al., 2021; Yehia et al., 2022). Fruit juices are abundant in antioxidants and include soluble substances such as sugars, organic acids, phenolic compounds, soluble pectin, and salts (Eraso-Grisales et al., 2022). Alternatively, these fruits are dehydrated (such as grape raisins) and added to baked products, snacks, or cereal breakfasts.

Considered as superfoods due to their high nutritional value and exquisite flavor, the production of cape gooseberries has expanded in Ecuador, but it has also encountered socioeconomic and productive obstacles. This crop is grown by the marginalized indigenous communities and small-scale farmers as a valuable source of daily income. This fruit is often wasted due to strict quality standards that discard the product with imperfections (INEN, 2009). In addition, there is any inspection or fresh products quality control at the market sites although the legislation is referring to free-of harm fresh products. This multifaceted problem affects the efficiency of the process and the availability for consumption or processing. In addition, rapid post-harvest deterioration limits the potential of the fruit market (Garavito et al., 2022). Bacteria and fungi are frequently found as agents of infection and microbiological spoilage of fruits after harvest (Lorenzo et al., 2018). Fruit consumption has been linked to foodborne disease outbreaks, which have been more frequently reported in recent years (Song et al., 2019). According to previous reports, *Botrytis cinerea* and *Alternaria alternata* are the most common spoilage microorganisms of cape gooseberry, reducing the shelf life to approximately two weeks at room temperature (Gonzáles-Aguilar et al., 2010). In addition, a significant number of enteric bacteria was detected in ready-to-eat cape gooseberries (Tenea et al., 2023). Food-associated pathogens are potentially hazardous because they may harbor genes for antibiotic resistance (Stec et al., 2022). At the national level, due to the lack of technology, these fruits are harvested manually from the field, transported to storage facilities, selected, and sent to the retail market. Additionally, by removing the calyx, the composition of bacteria on the fruit exocarp might change. However, 45% of the postharvest loss has been reported by the producers (Lagos-Burbano et al., 2020). Based on previous reports, several microbes have been associated with improper transportation, improper storage conditions, water rinsing, and human contact with contaminated hands (Leff and Fierer, 2013). Preliminary bacteriological analysis indicated the presence of *Enterobacteriaceae* that colonize cape gooseberry fruits purchased from the local market (Reyes et al., 2023). They can enter the fruit pulp and reduce its quality (Allard et al., 2020). Some microorganisms isolated from cape gooseberries demonstrated resistance to various antibiotics, thus, fresh fruits are a complex microenvironment suitable to transfer resistant microorganisms to humans (Tenea et al., 2023). In addition, antibiotic use during agricultural production may also have an impact on the environment pollution and the agricultural products (Stec et al., 2022). Despite of these limitation, this crop production is economically of interest due to the agroclimatic conditions and improvement of the varieties as well as does not involve any sophisticate technology. Although the production has favorable sociodemographic characteristics, such as the participation of relatively young agricultures and the inclusion of women in the production network (Moreno-Miranda et al., 2019), the production network require attention, the formation of the technical knowledge regarding post-harvest, transport logistics, manipulation and inappropriate storage that affect fruit safety and quality.

The microbiome assembly influences plant growth and health; however, there is little understanding of this process, especially concerning the microbiome of fruits (Olimi et al., 2022). The impact of microbial diversity on fruit quality and storage stability has been less studied. Fruit production and storage face many challenges from bacterial and fungal pathogens as well as other factors in different farming systems. A recent DNA metabarcoding approach to examine the microbial community differences between conventional and organic system pear fruit hypothesized that the microbial community’s variation during storage could be the cause of the postharvest quality and decay differences between the two types of fruits (Gao et al., 2023). Nonetheless, fruits include normal flora that is nonpathogenic and at the lesser extent pathogenic species (Balali et al., 2020). Enhancing the health and quality of pre- and post-harvest plants, as well as fruit quality, may be possible by using targeted methods to induce changes in the microbiome in plants and consequently in fruits. However, the rate of decay may increase or slow down due to the growth of microbial colonies on the fruit exocarp (Vermote et al., 2022). The customer safety is uncertain because of the increased risk of pathogenic microbe contamination resulting from the poor agricultural practices to postharvest handling to meet the increasing demand (Balali et al., 2020). The microbial diversity in cape gooseberry fruits at different stages of ripening might help track different microorganisms that colonize the fruit exocarp that can limit the fruit shelf life or help agriculture improve the quality of the fruit. Thus, this study aimed to measure the diversity and taxonomy of bacterial communities in cape gooseberry fruits collected from an organic farm production system in various stages of ripening using the 16S rRNA amplicon gene sequencing approach. The study compared the farm fruits to those from the local open-air market to track unexpected taxa (i.e. pathogens) with possible implications on fruit safety. Additionally, using conventional bacteriological analysis, total viable counts along with physicochemical characteristics were evaluated as preliminary fruit safety and quality indicators. The identification and quantification of epiphytic microflora associated to cape gooseberries exocarp could help to identify the critical points of contamination in the transition from agriculture field to table and constitute an essential step in developing measurements systems for preventing the microorganisms colonization and spread, and finally allowing strengthening the production and marketing chains of safe and better quality products.

## Materials and methods

### Sample collection experimental design and processing

**Figure 1:**
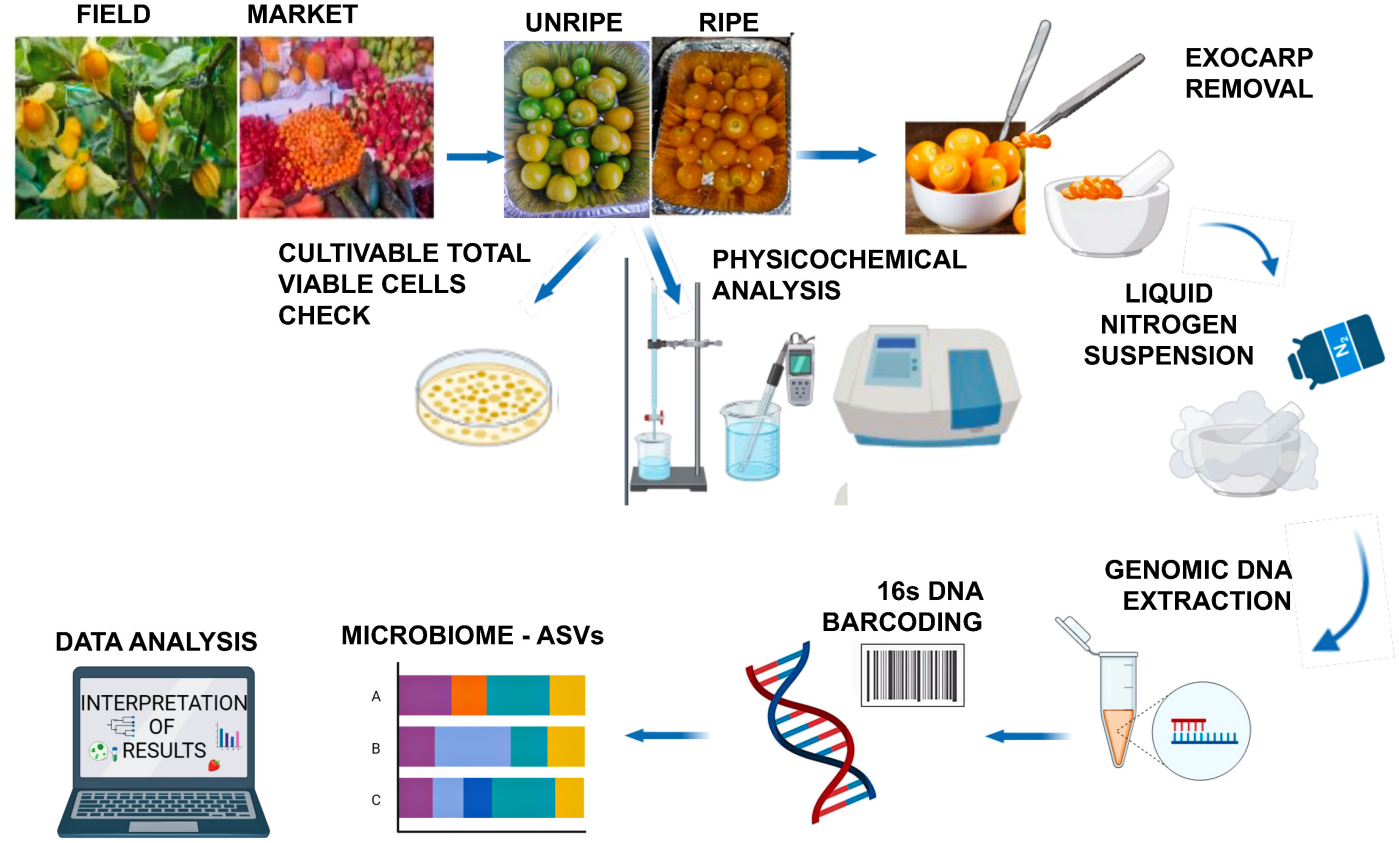
Workflow, processing, and analysis of cape gooseberries. (created with BioRender.com).

Cape gooseberries were harvested from an organic certified local producer in Imbabura Province (Parroquia San José de Quirquinche, 0°18′00″N 78°16′00″O) on May 2023. As an indicator of ripeness, the color of the calyx was considered (Oliveira Diniz and Coelho-Novembre, 2019). Fruits in unripe phase two with green calyx and green fruit, and phase four with straw-colored calyx and yellow fruit were randomly collected according to the following experimental design: 15 fruits (≈50 g) × field row × six field rows × two ripeness stages × three repetitions (total 540 fruits). **Supplementary Figure 1** illustrate the ripeness stages of the fruits employed in this study. Furthermore, fruits (ready-to-eat without calyx) were purchased from the retail market stands of Ibarra city (0°21′46″N 78°07′48″O) as follows: 15 fruits × six stands × three repetitions (total 270 fruits). The exocarp of each fruit was gently removed with a sterile knife placed in liquid nitrogen, finely ground in a prechilled mortar and pestle to obtain a homogenized mixture, and the mixture was further subjected to DNA extraction. Complementary analysis to detect the presence of total viable counts (TVC) in fruits was performed as previously described (Tenea et al., 2023). **Figure 1** shows an overview of the workflow process.

### Library construction, sequencing, and data processing

The MiSeq Sequencing System platform (paired-end 150 bp read length, Illumina, USA) was used for the genomic sequencing (custom design assay, Biosquence S. A., Quito, EC) using a thorough workflow that included a benchtop sequencing system, on-board primary analysis, and secondary analysis using MiSeq Reporter or BaseSpace (Illumina, USA). The 16S rRNA V3-V4 region was amplified with bacterial primers 341F (5’-CCTACGGNGGCWGCAG-3’) and 805R (5’-GACTACHVGGGTATCTAATCC-3’) (Klindworth et al., 2013), after which Illumina sequencing adapters and dual index barcodes were added to the target amplicon. As negative controls: 1) All DNA extraction and subsequent technique stages contained a blank extraction control. There were no input data for this empty control. 2) A control library that used DNA-free water as input for library formation and subsequent sequencing instead of using the extraction process. The FASTq files were subjected to a quality and filtering process to ensure taxonomic classification. For the analysis of marker gene-based microbiome sequencing data, the QIIME v.2 (Quantitative Insights into Microbial Ecology) pipeline version 2023.5 (Bolyen et al., 2019) was used. Sequences were clustered and denoised using the DADA2 method (Callahan et al., 2020). Higher-resolution tables of amplicon sequence variants (ASV) were generated with DADA2. Chimeric sequences were filtered using USEARCH 6.1 (Edgar, 2010). Sequences were assigned a taxonomy. A reference database containing sequences whose taxonomic composition was determined was also compared to that of the query ASV. Using a taxonomy classifier, the closest taxonomic affiliation with a certain degree of consensus or confidence was found based on the k-mer frequencies of the alignment. Other sequences may have different taxonomic annotations. Finding the closest match is not enough. Therefore, a Bayesian classifier was used to analyze the SILVA v132 (https://www.arb-silva.de/) reference taxonomy database for bacteria (Quast et al., 2013). 16S rRNA sequences derived from chloroplasts and mitochondria were not included in downstream analyzes.

### Rarefaction curves

Rarefaction curves were used to evaluate how the number of sequences (reads) analyzed affects a sample’s biological features or species diversity (Kandlikar et al., 2018). These curves provide insight into how sequencing depth affects the ability to fully capture a sample’s diversity.

### Alpha and beta diversity analysis

The analysis computes alpha diversity indices for each sample and compares the values of the indices between samples or groups to determine the variations in species composition within a given group or sample. The following metrics were calculated: Shannon’s diversity index: a quantitative measure of community richness and evenness; Observed features: a qualitative measure of community wealth; Faith’s phylogenetic diversity: a qualitative measure of community richness that incorporates phylogenetic relationships between traits; and evenness or Pielou uniformity: a measure of community equality. The results are shown as boxplots. The alpha diversity data were compared using a nonparametric Kruskal-Wallis test or one-way ANOVA on ranks to determine whether the samples were drawn from the same distribution. To quantify beta diversity, both nonphylogenetic methods (Bray–Curtis dissimilarity and Jaccard distance) and phylogenetic (UniFrac distance) were used. The sequences in the read data were sorted by lowest taxonomic level normalized for sampling depth square root transformed and a similarity matrix generated by calculating Bray–Curtis coefficients as reported by Lozupone et al. (2007). Weighted and unweighted UniFrac distance matrices and 999 Monte Carlo permutations were used to assess the statistical significance of the differences found. The composition of the bacterial communities was evaluated using principal coordinate analysis (PCoA) and permutational analysis of variance (PERMANOVA) using PRIMER6 and PERMANOVA+ (versions 6.1.12 and 1.0.2; Primer-E Quest Research Ltd. Auckland New Zealand). Heatmaps were constructed, and hierarchical clustering with the unweighted pair group method (UPGMA with Euclidean distance) was performed to determine the similarities or differences between the groups at the genus level. Venn diagrams were created to examine the intersection of the bacterial families and species between groups (two, four, and market). These analyzes were performed on a bioinformatics platform (https://www.bioinformatics.com.cn/en).

### Statistical significance tests

The Analysis of Similarities (ANOSIM) was used to test the hypothesis that there was no difference between two or more samples. This method uses permutation testing to identify similarities both within and between groups. The R test statistic was used to determine whether there were differences between the groups under the null hypothesis (Yinglin et al., 2018). Furthermore, analysis of microbiome composition (ANCOM) was used to identify taxa with significantly differential abundance to infer information about absolute abundance (Mandal et al., 2015). The compositionality of the microbiome data was considered using the centered log ratio (CLR) transformation. For a given taxa, the output W statistic shows the number of CLR-transformed models in which the taxon is differentially abundant considering the variable of interest.

### Evaluation of quality characteristics of fruits

Fruit samples from both fields (phases two and four) and market stands (ready to eat) were used to determine pH, total soluble solids (TSS), total titratable acidity (TTA), total polyphenol content (TPC), ascorbic acid content (AAC) and antioxidant capacity (AOX). A Mettler Toledo electrode immersion pH meter (S210 Columbus, OH USA) was used to measure the pH. A digital refractometer was used to measure the amount of TSS (AOAC, 2003). TTA was determined by titrating 25 ml of pulp juice obtained with 0.1 N NaOH and using phenolphthalein as an indicator (AOAC, 2003). The Folin–Ciocalteu method was used for TPC estimation using gallic acid (Sigma–Aldrich Co. LLC Saint Louis MO USA) as the standard (Singleton et al., 1999). A standard 26 dichloroindophenol titrimetric method was used to determine the vitamin C content (reduced ascorbic acid) (AOAC, 2003). AOX activity was determined by the 11-diphenyl-2-picrylhydrazyl (DPPH) method (Sigma-Aldrich Co. LLC Saint Louis, MO USA) (Rojas-Ocampo et al., 2021). All experiments were performed in triplicate. The results are reported as the mean ± standard deviation. Principal component analysis (PCA) of seven variables (pH, TSS, TTA, AAC, TPC, AOX and TVC) was conducted in all fruit samples of the three groups (field phase two, four, and market). Additionally, a Pearson correlation was performed to determine whether there was an interaction between the response variables.

## Results and discussion

### The structure of bacterial communities in cape gooseberry: alpha and beta diversity

**Figure 2:**
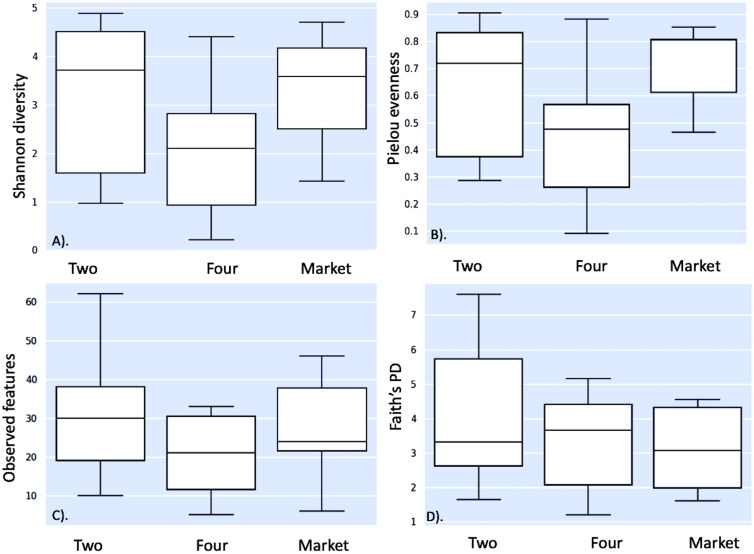
Microbial community structure: alpha diversity. Boxplots visualizing the results of the nonparametric Kruskal-Wallis test comparing **(A)** Shannon diversity index; **(B)** evenness; **(C)** observed features; and **(D)** Faith’s PD (phylogenetic diversity). Two: fruits collected from agricultural field phase two; Four: fruits collected from agricultural field phase four; Market: fruits purchased from market stands.

**Figure 3:**
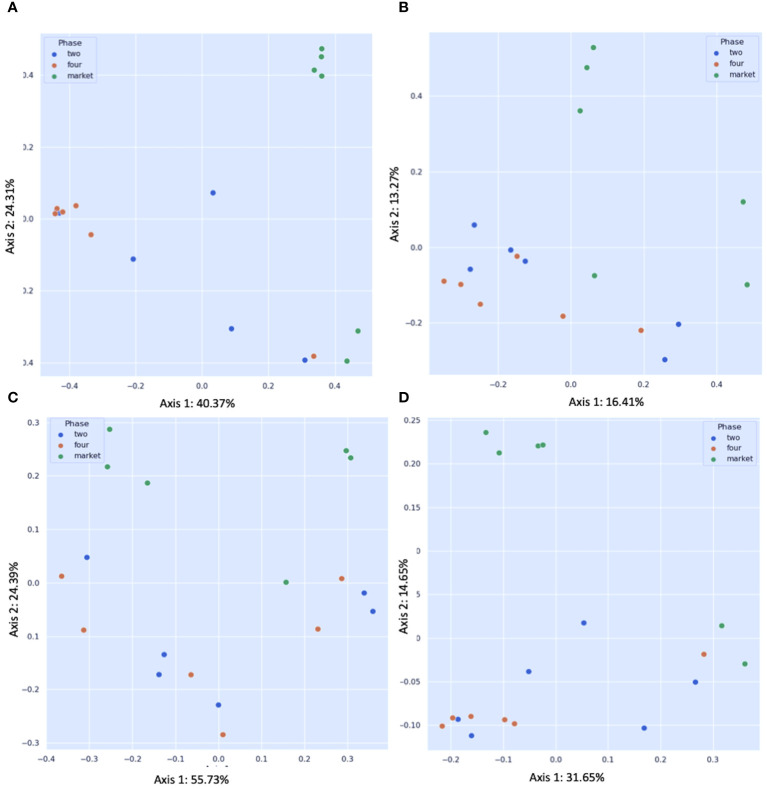
Principal coordinate analysis (PCoA) plots of beta diversity. Statistical significance between groups according to distance matrices for beta diversity: **(A)** Bray–Curtis dissimilarity indices; **(B)** Jaccard distance; **(C)** unweighted UniFrac distance; **(D)** weighted UniFrac distance. Statistics were calculated using pairwise PERMANOVA with 999 permutations. Blue dots: fruits collected from the field ripe phase two; orange dots: fruits collected from the field ripe phase four; green dots: fruits collected from the market stands.

This is the first microbiome analysis of cape gooseberry fruits in different maturity stages. The sequencing of bacterial communities by amplicon yielded an average of 231593.66 high-quality reads. The details of the non-chimeric input reads are shown in **Supplementary Table 1**. After filtering, to remove nontarget reads (e.g. chloroplasts or mitochondria), the sequences were assigned to ASVs (**Table 1**). Rarefaction analysis indicated that a sequencing depth of 1000 was sufficient to capture most taxa present in the samples (**Supplementary Figure 2**). When the curve is examined, it can be observed how the curve flattens when additional readings are gathered. In agreement with the ASV data, the Shannon diversity index revealed that fruits purchased from the market and the unripe stage had the highest level of bacterial diversity (average Shannon indices of 3.3 and 3.1, respectively) followed by fruits collected from the field in the mature (four) ripe stage (average Shannon indices of 2.07) (**Table 1**). A comparison between the bacterial diversity in each group (field vs. market) and its uniformity (Pielou uniformity) is shown in **Figures 2A, B**. Considering the origin of the samples (farm field and market), the Kruskal–Wallis test revealed no significant differences (H=1.67; p = 0.200) in alpha diversity between field phase four and the market, neither significant difference (H=2.08; P=0.149) between phases two and four (field); nonetheless, fruits collected from field phase four were less diverse than those collected from phase two and the market. There was no significant variation in alpha diversity between phase two and the market (H=0.00; p = 1.0). Furthermore, the microbial richness (observed ASVs) and Faith’s phylogenetic diversity (community richness) showed trends like those of microbial diversity, albeit with slight variations (**Figures 2C, D**). The alpha diversity analysis based on Shannon index values did not indicate significant differences in bacterial diversity between unripe and ripe fruits. Early metagenomic analysis on berries (strawberry) revealed no significant difference in bacterial diversity in ripe and stored fruits, while a significant difference was observed when analyzing different plant compartments (Olimi et al., 2022). The analysis of beta diversity indicates the extent of similarities and differences between microbial communities. We found significant differences between field phase four and market groups as shown by Bray–Curtis (pseudo-F = 6.215, p = 0.002; PERMANOVA) (**Figure 3A**) and Jaccard (pseudo-F = 2.253, p = 0.002; PERMANOVA) non-phylogenetic distance analyzes (**Figure 3B**). Furthermore, significant differences were detected by measuring the unweighted UniFrac distance as a phylogenetic index (pseudo-F = 3.198, p = 0.042; PERMANOVA) (**Figure 3C**). The market group samples formed a distinct group from the field samples regardless of their ripening stage, as shown in the PCoA map for the abundance unweighted UniFrac distance (**Figure 3C**). The variable F1 (Axis 1) explained 55.73% of the total variance by loading the samples from the market in the positive direction, while F2 (Axis 2) explained 24.69% by loading the samples from field settings (phases two and four). No significant differences in beta diversity were detected when the weighted UniFrac distance was measured (pseudo-F = 1.951, p = 0.068; PERMANOVA) (**Figure 3D**). Similarly, significant differences were observed in Bray–Curtis (pseudo-F = 3.688, p = 0.002; PERMANOVA) (**Figure 3A**) and Jaccard (pseudo-F = 1.796, p = 0.022; PERMANOVA) non-phylogenetic distances (**Figure 3B**), but not when using unweighted and weighted UniFrac distances as phylogenetic indices, when comparing field phase two and market groups. Likewise, no significant differences in beta diversity were observed between phase two and phase four of the field. Furthermore, based on the Bray-Curtis similarity analysis (ANOSIM) results, the R values between group four and the market, as well as between group two and the market, were close to 1.0 (0.79 and 0.69) suggesting dissimilarity between groups. Also, the R-value close to 0 (-0.01) suggests a uniform distribution between groups two and four originating from the field. These findings corroborated the previous profile of the bacterial community of strawberries showing that the microbiome of fruits purchased from the market is different from those originating in the field (Tenea and Reyes, 2024).

**Table 1:** Sequence characteristics and Shannon diversity index.

sample ID	Phase	Sequence counts	Filtered counts	Shannon diversity index	Total Species-level Taxonomic Categories Identified
U2L1	two	279482	204865	4.525	263
U2L2	two	205407	143999	2.968	247
U2L3	two	234158	162087	0.970	262
U2L4	two	227238	161224	4.886	385
U2L5	two	225012	148942	1.141	218
U2L6	two	196375	136772	4.450	329
U4FL1	four	177909	118622	1.573	199
U4FL2	four	233843	160125	0.718	288
U4FL3	four	252363	172347	2.875	381
U4FL4	four	199243	137881	0.209	320
U4FL5	four	222479	150348	4.406	269
U4FL6	four	306634	201731	2.627	379
UP1	market	214817	148195	2.162	302
UP2	market	210599	136413	1.420	351
UP3	market	218318	133561	4.351	424
UP4	market	298451	155104	4.707	548
UP5	market	226920	122913	3.638	432
UP6	market	239438	144965	3.535	439

U2L1-U2L6: fruits collected from the agricultural field unripe phase two; U4FL1-U4FL6: fruits collected from the agricultural field ripe phase four; UP1-UP6: fruits purchased from the market (ready-to-eat).

### Cape gooseberry bacterial diversity and taxa classification

**Figure 4:**
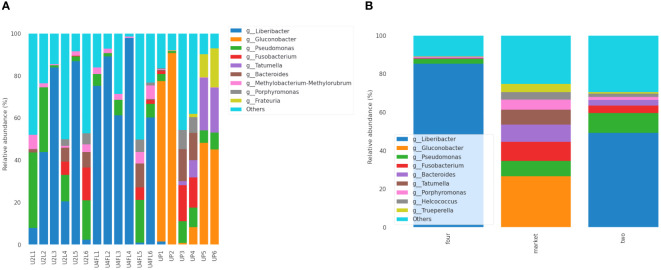
Relative abundance (%) of the bacterial communities at genus level identified in cape gooseberry: across the samples **(A)** and groups **(B)**. The stacked bar plots shows were constructed based on the relative abundance of the top 10 bacterial species, while “Other” category was defined as the sum of all classifications with less than 0.50% abundance. U2L1-U2L6: fruits collected from the agricultural field ripe phase two; U4FL1-U4FL6: fruits collected from the agricultural field ripe phase four; UP1-UP6: fruits purchased from market stands.

The diversity and abundance of the bacterial community may vary with the ripening stage, storage temperature, post-harvest handling, and fungicide treatment (Umar et al., 2023). In this study, the sequencing results showed that Proteobacteria, Firmicutes, Bacteroidota, Fusobacteria, and Actinobacteriota were the five most abundant phyla in all samples. Many rare phyla such as Desulfobacterota, Halobacterota, and Planctomycetota, were found at low relative abundance (< 1%) in samples originating from the field, while Chloroflexi was found in the market samples (< 1%). At the family level, a high diversity of microorganisms was found, with *Rhizobiaceae*, *Acetobacteraceae*, *Pseudomonadaceae*, *Fusobacteriaceae*, *Erwiniaceae, Peptostreptococcales-Tissierellales, Porphyromonadaceae*, and *Actinomycetaceae* being the most abundant across the samples (**Supplementary Figure 3A**). Among the groups, *Rhizobiaceae* was the most abundant family in fruits in the unripe (phase two) and ripe (phase four) stages, while *Acetobacteraceae*, *Fusobacteriaceae, Erwiniaceae*, and *Peptostreptococcales-Tissierellales* were the most abundant families in the market group (**Supplementary Figure 3B**). Additionally, 33 (17.1%) families were shared between the groups regardless of stage, while 10 (5.2%) and 11 (5.3%) were shared by the two- and four-market groups, respectively (**Supplementary Figure 3C**). Early metagenomic analysis of several Argentinian fruits revealed the presence of *Rhizobiaceae* in passion fruit in the ripe and almost ripe stages of maturity (Vermote et al., 2022). In the present study, species of the *Erwinia* genus were accounted at lower abundance (< 1%) on fruits originating from the market. *Erwinia* spp. are known as phytopathogens that cause fire blight in apples and pears (Junker and Keller, 2015). Previously, *Erwinia* spp. was detected in guava fruit samples (Vermote et al., 2022). Furthermore, the results indicated that the *Liberibacter* was the most abundant in the samples collected from the field (ripe two, four), while *Gluconobacter* was the most abundant in the samples collected from the market (**Figures 4A, B**). Moreover, the genus *Tatumella* was found exclusively in fruits purchased from the market, despite their variable abundance (1.99-3.97%) (**Figures 4A, B**). The two most common taxa, *Tatumella ptyseos*, and *T. saanichensis*, accounted for 4.28% and 13.56% of total reads. The proportion was sample-dependent. Previous studies have indicated that *Pantoea* and *Tatumella* are the most abundant bacterial taxa associated with resin canal discoloration in mango fruits (Umar et al., 2023). In addition, reports indicated that *Pantoea citrea* and *T. ptyseos* are the causes of pink pineapple disease (Marín-Cevada et al., 2010). Recent metagenomic analyses of ready-to-eat strawberries that were purchased on the same market as the cape gooseberry fruits used in this study revealed the presence of several enteric bacteria including *Enterococcus, Pantoea, Raoultella* and *Klebsiella aerogenes* (Tenea and Reyes, 2024). This suggests possible cross-contamination at the market stands; nonetheless, this assertion needs to be verified because at this point, we are unsure of the exact relationship between those taxa and market stands contamination. Additionally, the composition of bacterial species differed between the groups. Based on the abundance information of the main genera/species of all samples, a heat map was drawn to check whether the samples with similar processing were grouped (**Supplementary Figure 4**). Moreover, a total of 37 species (15.9%) were shared by all groups, regardless of the ripe stage, while 11 species (4.7%) and 9 species (3.9%), respectively, were shared by the two- and four-market groups (**Supplementary Figure 5**). Furthermore, the percentile of abundance feature by groups shows that *Candidatus*_Liberibacter and *Gluconobacter* spp. were the differential groups according to ANCOM analysis (**Supplementary Table 2**). The most abundant species was *Candidatus* Liberibacter in fruits originating from the field regardless of the ripe stage, while *Fusobacterium necrophorum, Porphyromonas levii*, *Helcococcus ovis, Trueperella pyrogens*, and *uncultured bacterium* in fruits originating from the market. *Candidatus* Liberibacter is a gram-negative bacterium, a phytopathogen that is an obligate parasite of the phloem of *Solanaceae* and citrus crops and is not cultivable *in vitro* (Reyes-Corral et al., 2021; Chen et al., 2022). This phytopathogen causes severe diseases to major crops worldwide (Batarseh et al., 2023). A recent report indicated that *Candidatus* Liberibacter causes yellowing and wilting in *P. peruviana* plants in Ecuador (Caicedo et al., 2020). In addition, was detected in *Physalis ixocarpa* Brot. (tomatillo) infections and causes a reduction in the quality and commercial value of the fruit on the market (Reyes-Corral et al., 2021). In this study, *Candidatus* Liberibacter was detected in the fruit surface of field samples, suggesting that might have a short lifespan as only the UP1 sample from the market contained (relative abundance of 1.6%) this phytopathogen. More temporal analyses are needed to corroborate this assumption. Bacterial colonization is influenced by location and season and the growth is favored by slow and sluggish fermentation and lack of oxygen protection (Sombolestani et al., 2019). High hexose concentrations, high acidity, tartaric acid, and sulfites create an environment that is selective for *Gluconobacter oxydans*, especially in the early stages of alcoholic fermentation (Sombolestani et al., 2019). More notable, the species *Frauteria aurantia*, was detected in the market samples UP3, UP5, and UP6 with a variable abundance of, 1.5%, 10.95%, and 18.45%, respectively. Interestingly, *F. aurantia* was detected in strawberry fruits purchased from the same local market (Tenea and Reyes, 2024). We stated that various environmental storage conditions including temperature and location of fruits in proximity to mummified fruits and vegetables could be linked to these cross-contamination events. Additionally, a phylogenetic tree derived from ASVs was constructed to reveal biological diversity and evolutionary relationships within the sequence data set. The position of the most abundant taxon according to its origin of sampling (filed vs. market) is shown in **Supplementary Figure 6**. However, there is still much to learn about the microbiome during post-harvest storage, particularly those responsible for fruit decay and post-harvest quality.

### Potential animal pathogens linked to cape gooseberry fruits

**Figure 5:**
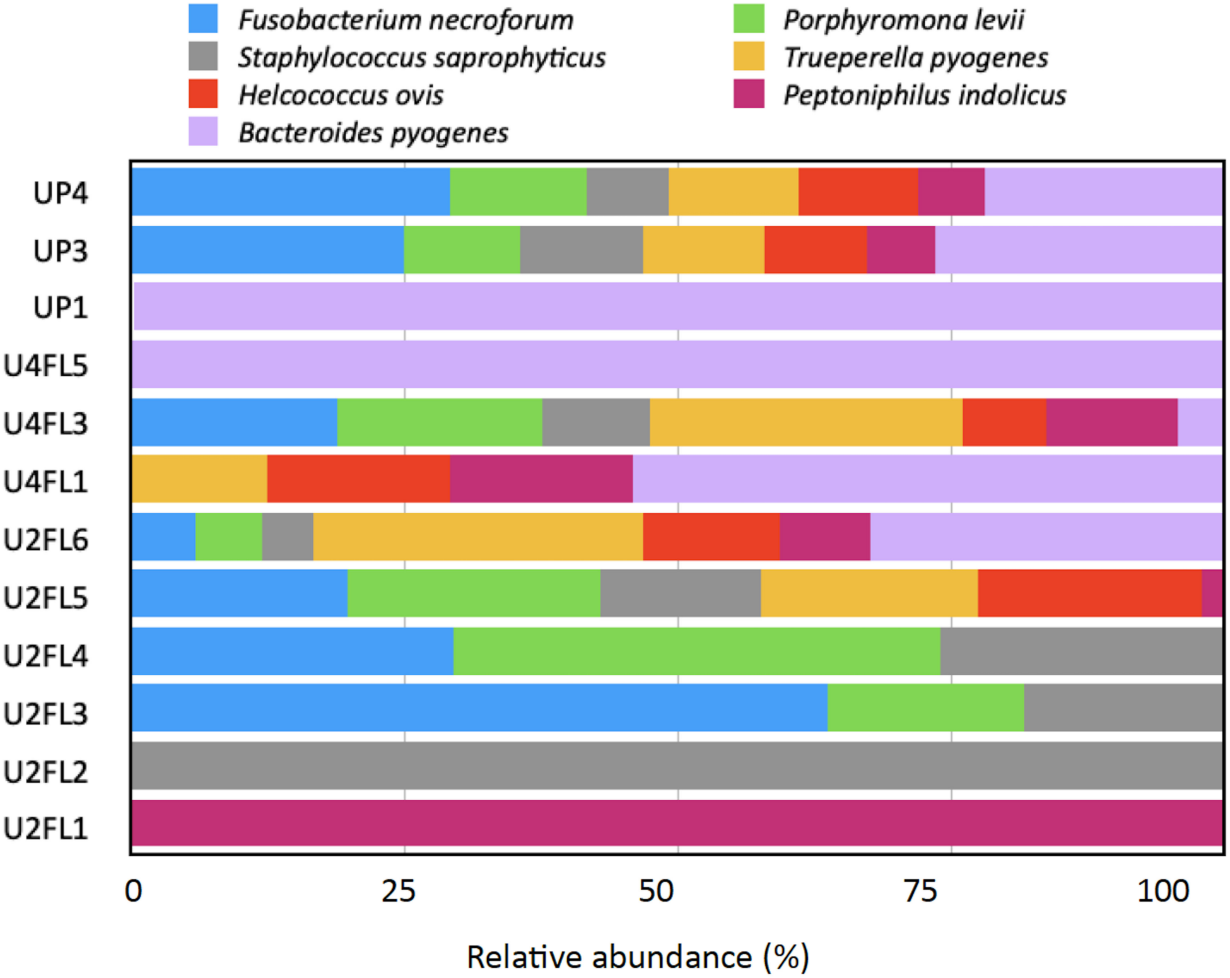
Relative abundance (%) of pathogenic species detected in cape gooseberry. U2L1-U2L6: fruits collected from agricultural field ripe phase two; U4FL1-U4FL6: fruits collected from agricultural field ripe phase four; UP1-UP6: fruits purchased from market stands.

Fruits and vegetables can be contaminated by pathogenic microorganisms both before harvest (soil, seeds, irrigation water, urine from domestic and wild animals) and after harvest (storage, preparation, and packaging) at multiple points in the supply chain (Aiyedun et al., 2021). These pathogens can adapt to a variety of environments because they have developed multiple strategies for effective attachment, survival, and colonization. The plant microbiome phyllosphere and plant responses are additional risk factors in a competitive environment; they both directly affect the pathogen’s ability to survive on the surface of the leaf (Thomas et al., 2024). In this study, the sequencing results revealed the presence of several potential pathogens in the samples regardless of their origin. However, with variable relative abundance, the bacterial species *Fusobacterium necrophorum, Porphyromonas levii*, *Staphylococcus saprophyticus*, *Bacteroides pyogenes*, *Helcococcus ovis*, and *Trueperella pyogenes* were detected in almost all samples (**Figure 5**). *Fusobacterium necrophorum* is an anaerobe, pleomorphic, gram-negative rod regarded as a commensal of the upper respiratory gastrointestinal and genital tracts in humans and animals (Creemers-Schild et al., 2014). *Helcococcus ovis* was detected in ovine mastitis and bovine valvular endocarditis (Kutzer et al., 2008) as well as in dairy milk obtained from a caw with puerperal metritis (Locatelli et al., 2013). *B. pyogenes* is known as an opportunistic pathogen and is a part of the skin biota and mucous membranes of the upper respiratory gastrointestinal or urogenital tracts in animals (Rzewuska et al., 2019). This pathogen causes a variety of purulent infections such as metritis mastitis pneumonia and abscesses, which in livestock breeding generate significant economic losses (Rzewuska et al., 2019). Furthermore, *B. pyogenes* was the most common bacteria associated with foot cattle lesions (Walker et al., 2023). In addition, two species of the genus *Peptostreptococcus, Peptoniphilus indolicus* and *P. levii*, were detected in fruits originating from the UP3 and UP4 market stands. These species are anaerobic gram-positive cocci and were detected in patients with pneumonia soft tissue infections or diseases of the colon or bladder (Brown et al., 2014). Additionally, *P. levii* was detected in dairy cattle with digital papillomatous dermatitis (Moe et al., 2010). Given that the fruits were harvested from an organic agricultural system that emphasizes crop rotation, companion planting (plants grown jointly with corn cultivars), and the use of organic fertilizers such as compost manure, green manure, and bone meal, we suggest that the pathogens may have spread from the soil to the leaves and eventually to the fruits. In addition, animals are a common source of contamination for enteric pathogens (Thomas et al., 2024). They can act as vectors, spreading pathogens from one location to another, or as the source of contamination through their excrement, which can be deposited in the soil, water, or directly onto the foliage. Although this is most likely the least controllable scenario (Thomas et al., 2024). A recent study conducted in Ecuador found that irrigation water systems may be the source of beta-lactam-resistant *Escherichia coli*, with 58% of 165 *E. coli* laboratory cultures exhibiting the ESBL (Extended Spectrum Beta-Lactamase) phenotype and 11% of the sampled vegetables including cape gooseberries testing positive for the bacteria (Montero et al., 2021). We found antibiotic-resistant *E. coli* in the cape gooseberries that were purchased from the market in a previous conventional bacteriological analysis (Tenea et al., 2023). According to the results of the current investigation, the samples obtained from the market stands contained traces of *K. variicola, Enterococcus faecalis, Escherichia vulneris*, and *Klebsiella pneumoniae* (each taxon <1%). As these fruits are handled by agricultural farmers (from the field to the wholesale collection center), manually selected (removing the calyx, sorted by size) stored at room temperature for about two days before being transported to the retail market, these conditions could let the existing pathogen survive and colonize. The microbial presence of hundreds of street market food samples, including fruits, from Ecuador, was surveyed by Salazar-Llorente et al. (2021) and Cárdenas et al. (2024). Their results point to inadequate sanitation and a shortage of clean water for food processing. More research is needed to understand the mechanisms underlying the relationships between plants and enteric pathogens, as well as how to mitigate contamination. Strict hygiene and sanitation protocols must be applied throughout the supply chain, from farm to fork, to prevent enteric pathogens from colonizing fruits. This will help develop evidence-based policies that will reduce the frequency of foodborne illness outbreaks.

### Cape gooseberry physicochemical properties

**Figure 6:**
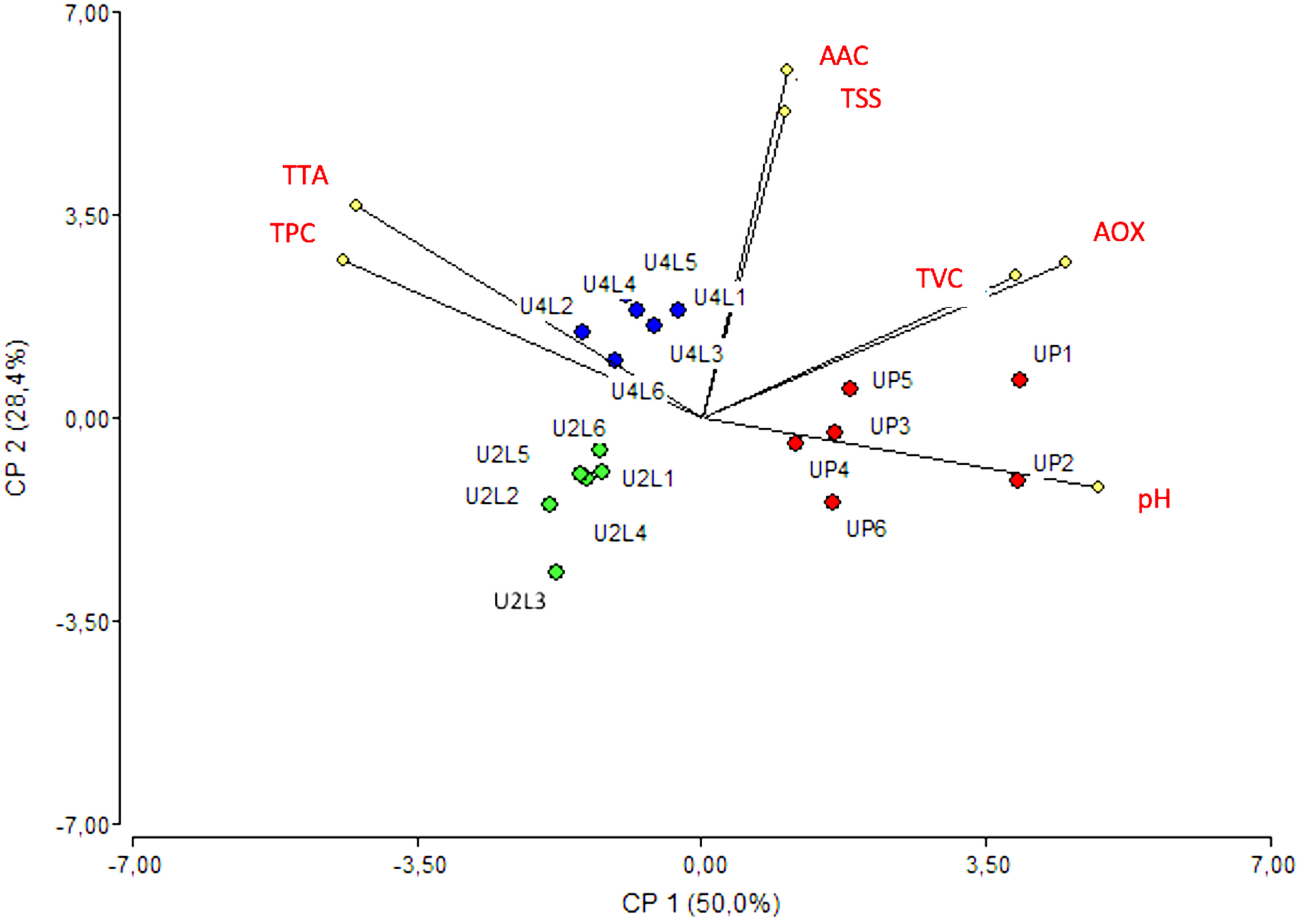
Biplot PCA analysis of cape gooseberry quality variables. The colors mark the closely related samples registered for each variable. U2L1-U2L6: fruits collected from agricultural field ripe phase two; U4FL1-U4FL6: fruits collected from agricultural field ripe phase four; UP1-UP6: fruits purchased from the market; TTA, Total titratable acidity; TSS, Total soluble solids; AOX, antioxidant capacity; AAC, acid ascorbic content; TPC, Total polyphenol content; TVC, Total viable cells.

The maturity of fruits influences their physicochemical and functional traits; thus, for excellent organoleptic and nutritional qualities, cape gooseberries must be harvested 35 days after anthesis (Gonzáles-Aguilar et al., 2010). The fruit microbiota can benefit or be detrimental to fruit quality. The growing number of current outbreaks caused by enteric pathogenic bacteria present in fresh products is of concern (Aiyedun et al., 2021). Pathogenic bacteria can alter the quality of fruit, resulting in both obvious and subtle alterations that can affect the texture, taste, safety, and appearance of the fruit. The physical and biochemical properties of plant surfaces can also influence the level of contamination because they can serve as a defense against bacterial pathogens. However, the calyx of cape gooseberry can preserve both safety and quality during storage (Novoa et al., 2006). In this study, standard culturing methods indicated that the fruits of the market had a greater number of viable cells (8.72 × 10^2^ UFC/g) varying with the purchase stand, while the fruits from the field showed a lower number of viable cells (6.33 × 10^1^ UFC/g: phase two, and 4.72 × 10^2^ UFC/g: phase four). Thus, the increase in viable cells in fruits collected from the market suggests that the hand removal of calyx as well as the inappropriate storage increase their susceptibility to contamination. The PCA of the seven variables (pH, total soluble solids, total titratable acidity, total polyphenol content, ascorbic acid content, antioxidant capacity, and total viable cells) revealed a clear separation of the samples according to their stage of ripening and origin (field vs. market). Fruits in the ripe stage (four) of the field were identified by their high polyphenol and titratable acidity, while the fruits in phase two displayed a distinct cluster that was far from all other vectors, indicating that their physicochemical properties were divergent. Fruits purchased from the market were distinguished by their high antioxidant capacity, pH, and viable cell counts (**Figure 6**). This discrepancy may be caused by the fact that fruits harvested from the field were processed immediately, whereas we are unsure of the length of time fruits bought from market stands were kept at room temperature. However, the variable pH showed a strong correlation with the total amount of soluble solids and the ascorbic acid content (0.83 and 0.99) according to the Pearson coefficient test. Furthermore, moderate correlations (pH = 0.54 and AOX = 0.62) were found between total viable cells and antioxidant capacity and between antioxidant capacity and pH. A high inverse correlation between pH and total polyphenols content was also found with a value of -0.78. This implies that the total polyphenol content decreases at high pH, which is consistent with the findings of other studies showing that the quality of the fruit is predicted by the polyphenol content (Kusstatscher et al., 2020). These results are in line with previous metagenomic studies in fruits (Zhang et al., 2011), suggesting that the microbiome might influence the quality attributes of the fruit.

## Conclusions

This is the first report on the microbiome associated with cape gooseberry fruits. The bacterial communities vary with the ripening stage and origin (field vs. market). The alpha diversity analysis did not show any significant differences in the diversity of bacteria within the samples, nonetheless, differences between the groups were seen by the beta diversity analysis. The phytopathogen *Candidatus* Liberibacter was abundant in fruits originating from the field, whereas potential animal pathogens *Fusobacterium necrophorum, Porphyromonas levii*, *Helcococcus ovis*, and *Trueperella pyogenes* were the most abundant in fruits originating from the market. In addition, some enteric pathogens were found in fruits purchased from the market only suggesting the contamination at the market stands. These results provide the first baseline data on the diversity and composition of the bacterial diversity in cape gooseberries. Fruit safety may thus be impacted by potential contamination by bacterial pathogens of animal origin in both field and market stands; this should be further investigated as any metagenomic study so far relates pathogens composition, toxicity and impact on human health. These scientific findings supporting the cross-contamination at market stands may assist authorities in taking action to increase both growers’ and sellers’ awareness. Nevertheless, more protocols to prevent contamination are required considering the overwhelming evidence of how raw foods become contaminated after harvest.

## Data availability statement

The data sets presented in this study can be found in online repositories. The names of the repositories and accession numbers can be found below: https://www.ncbi.nlm.nih.gov/sra/PRJNA1070422.

## Author contributions

GNT: Conceptualization, Data curation, Formal analysis, Funding acquisition, Investigation, Methodology, Project administration, Software, Supervision, Validation, Visualization, Writing – original draft, Writing – review & editing. DM: Formal analysis, Investigation, Writing – review & editing.
