# Supplementary material for: Bacterial community structure of Physalis peruviana L. fruit exocarp and the presence of pathogens with possible implications on food safety
Source: Front Plant Sci. 2024 Jul 18;15:1410314. doi: 10.3389/fpls.2024.1410314 (PMC11291218; doi:10.3389/fpls.2024.1410314)

Supplementary Material

Bacterial Community Structure of *Physalis peruviana* L. fruit Exocarp and the Presence of Pathogens with Possible Implications on Food Safety

**Gabriela N. Tenea*, Diana Molina**

Biofood and Nutraceutics Research and Development Group, Faculty of Engineering in Agricultural and Environmental Sciences, Technical University of the North. Av. 17 de Julio s-21 y José María Córdova. Barrio El Olivo, 100150 Ibarra, Ecuador.

*****Correspondence: gntenea@utn.edu.ec;

**Supplementary Table 1.** Filtered reads of each sample obtained via DADA2 statistics.

| **Sample ID** | **Input reads** | **Filtered reads** | **Input passed filter (%)** | **Denoised** | **Merged** | **Input merged (%)** | **Non-chimeric reads** | **Input non-chimeric reads (%)** |
| --- | --- | --- | --- | --- | --- | --- | --- | --- |
| **U2L1** | 279482 | 210459 | 75.3 | 208527 | 206516 | 73.89 | 204865 | 73.3 |
| **U2L2** | 205407 | 149919 | 72.99 | 147455 | 145444 | 70.81 | 143999 | 70.1 |
| **U2L3** | 234158 | 167823 | 71.67 | 165742 | 163834 | 69.97 | 162087 | 69.22 |
| **U2L4** | 227238 | 166902 | 73.45 | 165052 | 162703 | 71.6 | 161224 | 70.95 |
| **U2L5** | 225012 | 155029 | 68.9 | 152680 | 149935 | 66.63 | 148942 | 66.19 |
| **U2L6** | 196375 | 142527 | 72.58 | 140192 | 137861 | 70.2 | 136772 | 69.65 |
| **U4FL1** | 177909 | 125539 | 70.56 | 122781 | 119779 | 67.33 | 118622 | 66.68 |
| **U4FL2** | 233843 | 166700 | 71.29 | 164409 | 161980 | 69.27 | 160125 | 68.48 |
| **U4FL3** | 252363 | 179892 | 71.28 | 177298 | 174341 | 69.08 | 172347 | 68.29 |
| **U4FL4** | 199243 | 143443 | 71.99 | 141354 | 139442 | 69.99 | 137881 | 69.2 |
| **U4FL5** | 222479 | 157541 | 70.81 | 155030 | 152145 | 68.39 | 150348 | 67.58 |
| **U4FL6** | 306634 | 212763 | 69.39 | 209645 | 205802 | 67.12 | 201731 | 65.79 |
| **UP1** | 214817 | 153917 | 71.65 | 152201 | 150145 | 69.89 | 148195 | 68.99 |
| **UP2** | 210599 | 143261 | 68.03 | 140527 | 138323 | 65.68 | 136413 | 64.77 |
| **UP3** | 218318 | 139843 | 64.05 | 137921 | 136026 | 62.31 | 133561 | 61.18 |
| **UP4** | 298451 | 165087 | 55.31 | 162082 | 158558 | 53.13 | 155104 | 51.97 |
| **UP5** | 226920 | 129186 | 56.93 | 128125 | 126663 | 55.82 | 122913 | 54.17 |
| **UP6** | 239438 | 151910 | 63.44 | 149961 | 147627 | 61.66 | 144965 | 60.54 |

Legend: U2L1-U2L6: fruits collected from agricultural field phase two; U4L1-U4L6-fruits collected from agricultural field phase four; UP1-UP6: fruits purchased from market (ready-to-eat).

**Supplementary Table 2.** Percentile abundances of features by group (ANCOM analysis).

| **Percentile** | **0** | **25** | **50** | **75** | **100** | **0** | **25** | **50** | **75** | **100** | **0** | **25** | **50** | **75** | **100** |
| --- | --- | --- | --- | --- | --- | --- | --- | --- | --- | --- | --- | --- | --- | --- | --- |
| **Group** | four | four | four | four | four | market | Market | market | market | market | two | two | two | two | two |
| ***Gluconobacter* spp.** | 1.0 | 1.0 | 1.0 | 1.0 | 1.0 | 1.0 | 68.0 | 974.75 | 1288.0 | 1445.25 | 1.0 | 1.0 | 1.0 | 1.0 | 1.0 |
| ***Candidatus_*Liberibacter** | 4 | 373.75 | 548.5 | 892 | 5288 | 1.0 | 1.0 | 1.0 | 1.0 | 31 | 28 | 39.5 | 630.5 | 1113.25 | 3431 |

**Supplementary Figure 1**. Illustration of the cape gooseberries ripeness stages employed in this study. Legend: F2: phase two; F4: phase four; Market: fruits from the market.

**Supplementary Figure 2.** Illustration of rarefaction curves determined for all strawberry samples (sequencing depth 1000x). Legend: U2L1-U2L6: fruits collected from agricultural field phase two; U4FL1-U4FL6-fruits collected from agricultural field phase four; UP1-UP6: fruits purchased from market stands.


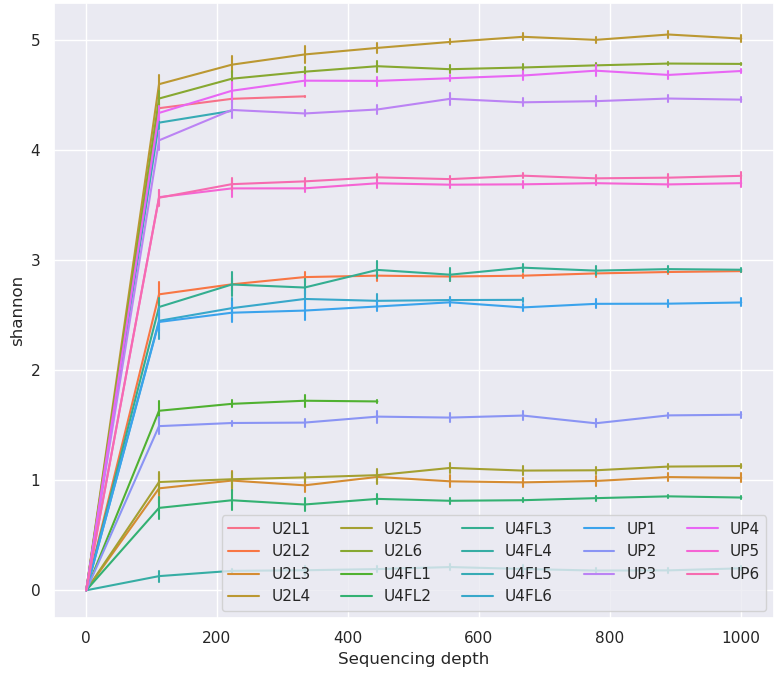


**Supplementary Figure 3.** Bacterial families across the samples (A) and groups (B) identified in cape gooseberry. The stacked bar plots shows were constructed based on the relative abundance of the top 10 bacterial families, while "Other" category was defined as the sum of all classifications with less than 0.50% abundance. C) Venn diagram showing the number and percentage of shared bacteria between the groups. Legends: U2L1-U2L6: fruits collected from the agricultural field ripe phase two; U4FL1-U4FL6: fruits collected from the agricultural field ripe phase four; UP1-UP6: fruits purchased from market stands.


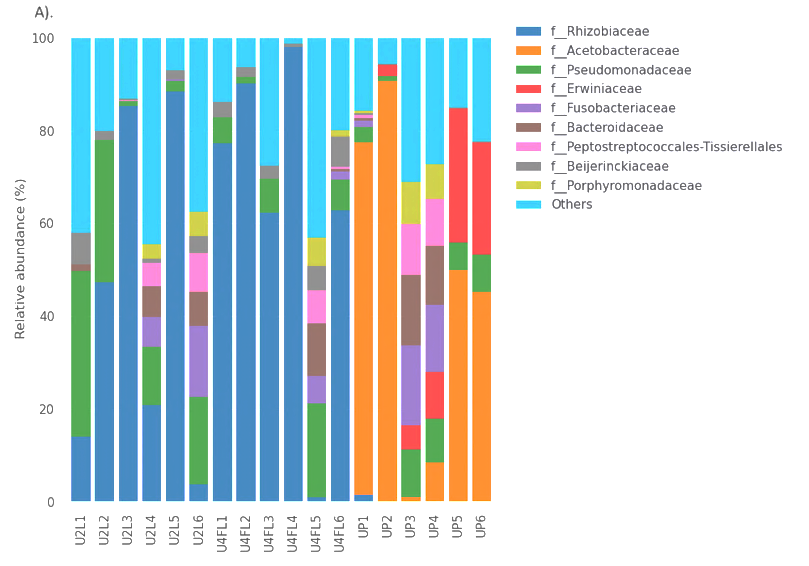

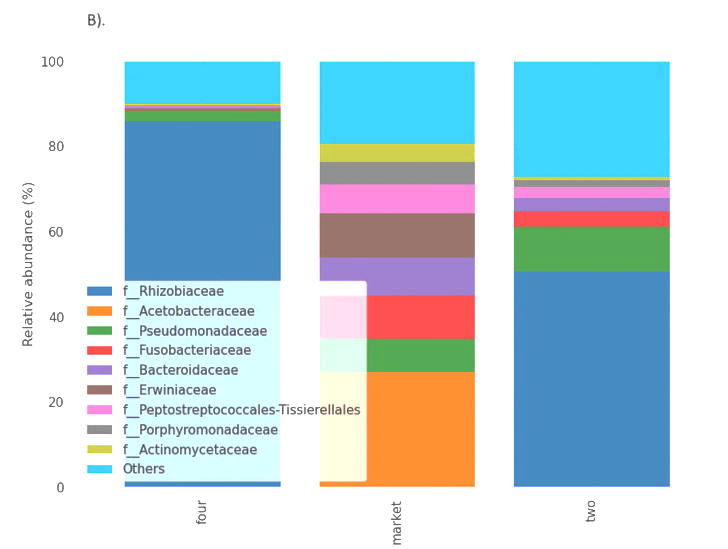


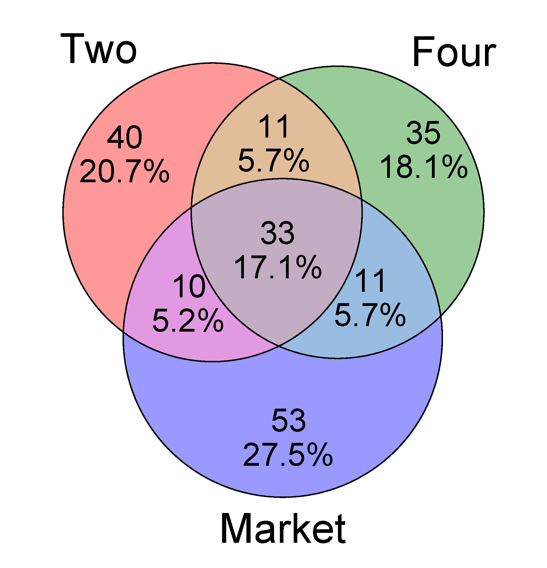


C).

**Supplementary Figure 4.** Heatmap and hierarchical clustering of the most abundant bacteria at the genus level. X-axis contains the microbial genus that have been identified in the samples; On the Y axis are the different samples and experimental conditions that are being compared (groups). The colors in the heat map represent the relative abundance of each microbial taxon in each sample, warmer colors represent a high abundance of these microorganisms in a sample, while darker colors indicate low abundance. Color bars: green: samples from the market; Blue: samples from the field, phase two; orange: samples from the field phase four.

**
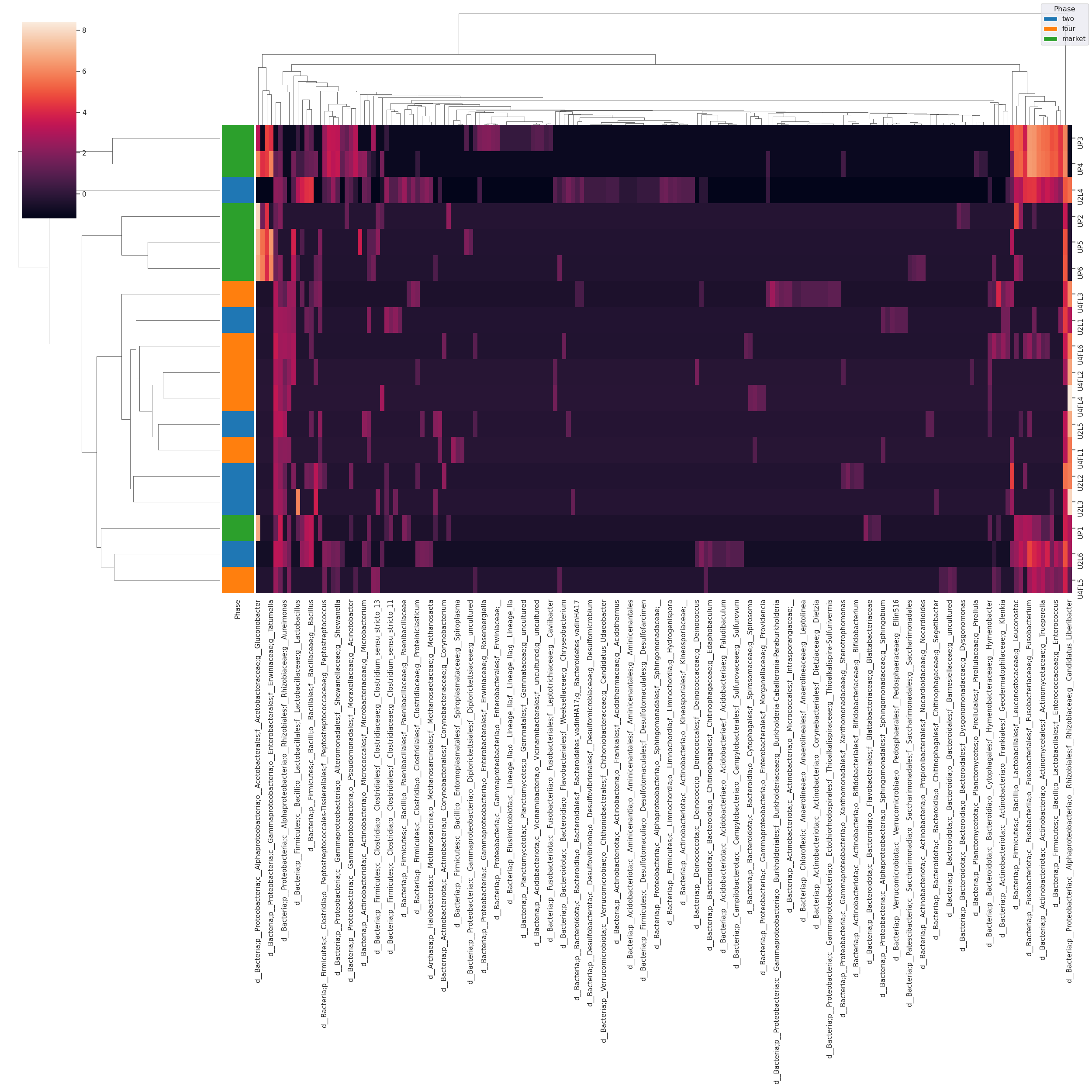
**

**Supplementary Figure 5.** Venn diagram showing the number and percentage of shared bacteria species between the groups.

**
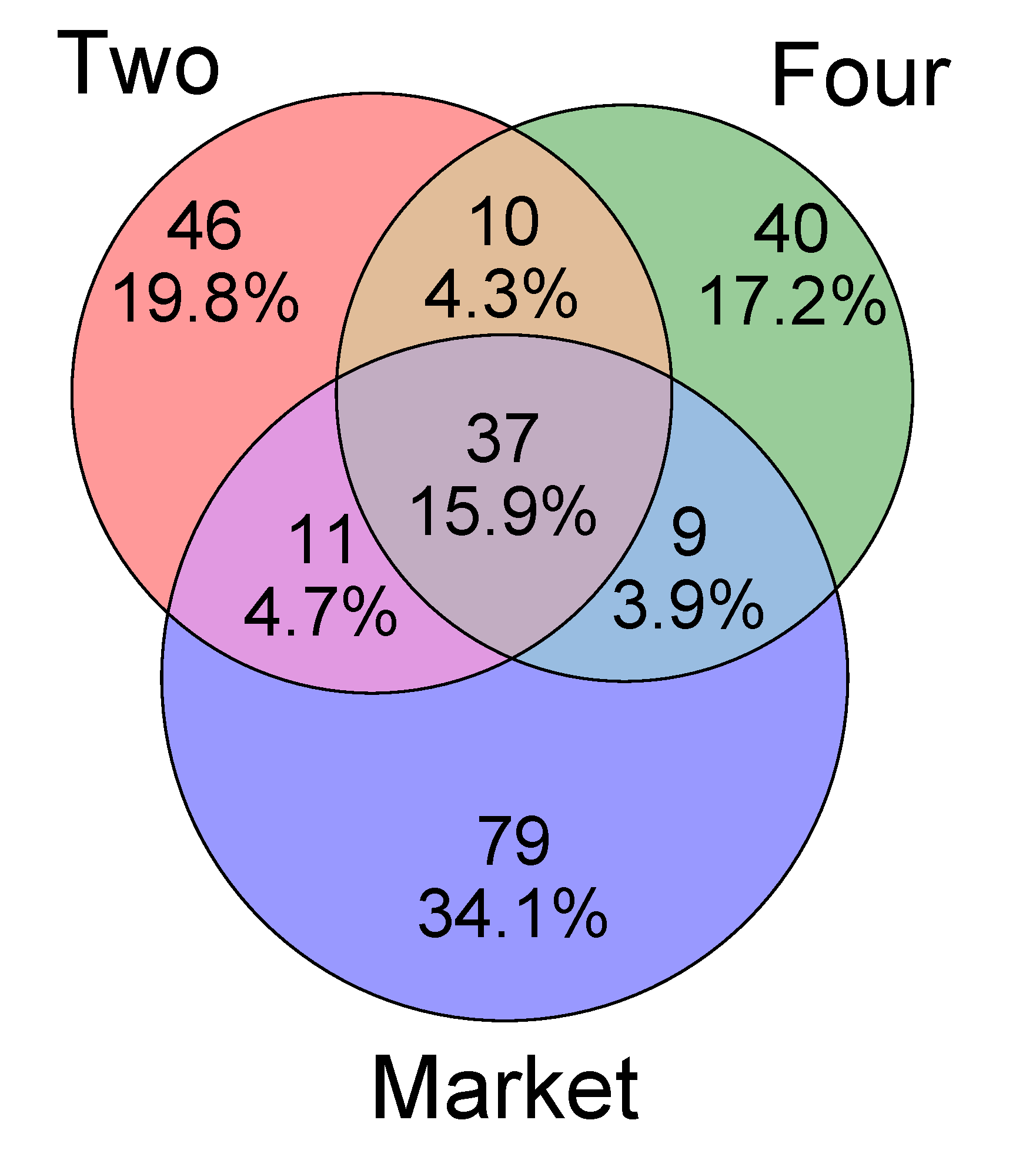
**

**Supplementary Figure 6**. Phylogenetic tree derived from 16S rRNA gene sequence data showing the position of the most abundant bacterial taxon. The color nodes in the tree are features (taxonomic annotation at the genus level). Internal nodes where all the descendants have the same feature metadata value are considered to have that value. Bar plots are the group metadata.


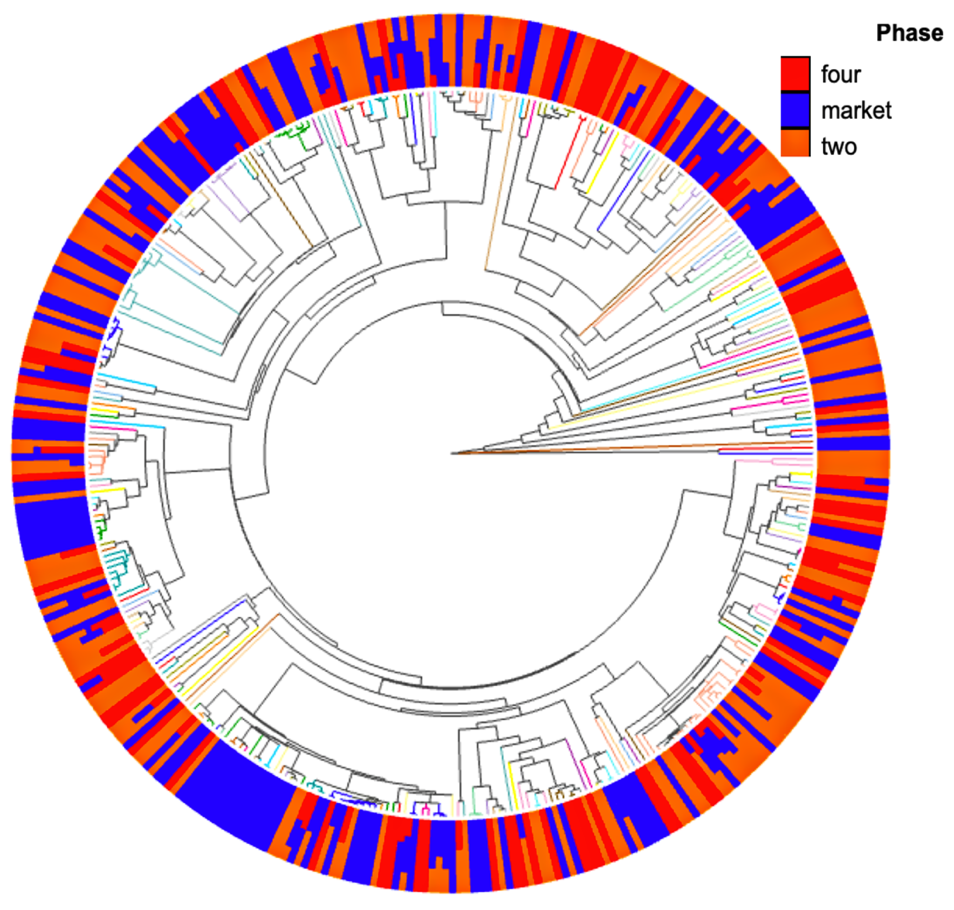

Supplement: Supplementary file 1 [file DataSheet_1.docx]
